# Determination of the compositions of the DIGM zone in nanocrystalline Ag/Au and Ag/Pd thin films by secondary neutral mass spectrometry

**DOI:** 10.3762/bjnano.7.41

**Published:** 2016-03-22

**Authors:** Gábor Y Molnár, Shenouda S Shenouda, Gábor L Katona, Gábor A Langer, Dezső L Beke

**Affiliations:** 1 Department of Solid State Physics, University of Debrecen, P.O. Box 2., Debrecen, 4010, Hungary; 2 Department of Physics, Faculty of Education, Ain Shams University, Cairo, Egypt

**Keywords:** diffusion-induced grain boundary migration, grain boundary diffusion, nanocrystalline films

## Abstract

Alloying by grain boundary diffusion-induced grain boundary migration is investigated by secondary neutral mass spectrometry depth profiling in Ag/Au and Ag/Pd nanocrystalline thin film systems. It is shown that the compositions in zones left behind the moving boundaries can be determined by this technique if the process takes place at low temperatures where solely the grain boundary transport is the contributing mechanism and the gain size is less than the half of the grain boundary migration distance. The results in Ag/Au system are in good accordance with the predictions given by the step mechanism of grain boundary migration, i.e., the saturation compositions are higher in the slower component (i.e., in Au or Pd). It is shown that the homogenization process stops after reaching the saturation values and further intermixing can take place only if fresh samples with initial compositions, according to the saturation values, are produced and heat treated at the same temperature. The reversal of the film sequence resulted in the reversal of the inequality of the compositions in the alloyed zones, which is in contrast to the above theoretical model, and explained by possible effects of the stress gradients developed by the diffusion processes itself.

## Introduction

It is known that during interdiffusion in micro- or nanocrystalline samples at low temperatures grain boundary (GB) diffusion-induced grain boundary migrations (DIGM) can be observed [1–5], during which an alloyed zone is left behind [1,4,6]. This alloying was one of the driving forces to investigate the phenomenon of DIGM, since it offered a controllable way to fabricate unique devices [7]. The driving force for DIGM can be different depending on the role of bulk diffusion and thus the following classification is plausible: i) a low-temperature regime in which the bulk diffusion is completely frozen and ii) intermediate-temperature regimes when there is a bulk penetration of the diffusing elements into the bulk (for several nanometers or deeper) ahead of the migrating boundaries. In the second case the elastic stress, created by the size mismatch of the two types of atoms in this zone can be the dominant contribution to the driving force [2,8]. Driving forces of the same origin can also be responsible for the formation of new grains with distinctly different compositions from the matrix: this is the diffusion-induced recrystallization (DIR) [9]. On the other hand it is also quite widely accepted that, at low temperatures (regime i)) the inequality of the GB diffusion fluxes leads to the migration of GBs [3–4,10].

Nevertheless the question about the composition left behind the sweeping boundaries in both cases is still not fully answered. Penrose [8] developed a general mathematical treatment for the regimes ii) and demonstrated that the steady-state solution of the problem requires a constant composition level behind the moving front. However, a prediction for the concentration level was not provided (see also the comment in [11]). For regime i) in [4], based on an atomistic model of the boundary shift by motion of GB steps, it was obtained that in an A/B diffusion couple (*J*_A_ > *J*_B_, i.e., *D*_A_ > *D*_B_*,* where *D_i_* denote the GB diffusion coefficients) the composition of A in the DIGM zone on the slower side (i.e., on the B side, with the initial composition *c*_A0_) can be given as

[1]
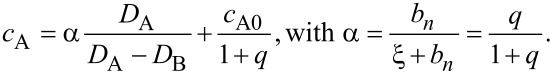


Here ξ and *b_n_* and are the step height in the GB and the Burgers vector component (perpendicular to the GB plane) of the GB dislocation (GBD) belonging to this step, respectively. Since the ratio *D*_A_/(*D*_A_ – *D*_B_) is slightly higher than unity the role of the multiplying factor, α, is important in determining the composition. The ratio *q* = *b_n_*/ξ was estimated to be of the order of 0.1 in [4] and thus the experimental values on the slower side were reproduced. In addition, in the literature mainly the alloying in the slower component (in B) was investigated and only few results on alloying on the other (faster) side can be found [12–13]. In a more recent series of experimental investigations in our laboratory [14–20] it was indicated that the compositions in the DIGM zones can be determined by secondary neutral mass spectrometry (SNMS) depth profiling. It was shown that i) as it was illustrated in the Cu/Ni system already in 1983 [21], alloying can also take place in the faster component, similarly as in the slower one of the Ag/Au system [16] and ii) if the grain size is less than half of the average sweeping distance of the GBs, *L*, a full homogenization is possible, with a saturation at compositions determined by the concentrations left behind the sweeping boundaries.

In addition it can be shown, by similar considerations as in Equation 1, that the composition of B left behind the moving boundary in A (faster side) can be given as:

[2]
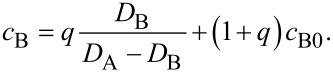


Thus from the investigations of the temperature dependence of the saturation values one can get estimations on the *D*_A_*/D*_B_ ratios on both sides of the diffusion couple.

In order to get clear conclusions about the validity of above predictions it is plausible to make a distinction between systems forming solid solutions and systems forming compound. In the latter cases the DIGM drives the system to phase equilibrium, dictated by the bulk phase diagram [19–20], and the description of these systems should go beyond, e.g., the theoretical model of [4], providing the relations in Equation 1 or Equation 2. On the other hand in systems forming solid solutions, according to Equation 1 and Equation 2, the saturation values should be different on the two sides of the couple, predicting a higher value in the slower component (in B). Indeed in our recent paper on Ag/Au nanocrystalline thin films at 150 °C [16] it was obtained that the saturation level of Au in Ag was lower than that of Ag in Au.

In this communication we report experimental results on the temperature dependence of the above saturation values in Ag/Au and Ag/Pd nanocrystalline thin film systems having complete mutual solid solubility at low temperatures (regime i)).

## Experimental

Nanocrystalline Ag(15 nm)/Au(15 nm)/substrate, Au(15 nm)/Ag(15 nm)/substrate, Ag(15 nm)/Pd(15 nm)/substrate, Pd(15 nm)/Ag(15 nm)/substrate, as well as Pd(30 nm)/Ag(30 nm)/substrate thin film systems were produced by magnetron sputtering onto single crystalline Si substrates with native SiO_2_ layers at room temperature. The base pressure in the sputtering chamber was below 2 × 10^−5^ Pa. During the deposition, the Ar (99.999%) pressure was 5 × 10^−1^ Pa under dynamic flow. The deposition rates were 0.573 nm/s and 0.868 nm/s for Pd and Ag, respectively. The Ag/Au samples were annealed under 1 bar Ar pressure at different temperatures (120–200 °C) for different annealing times. The Ag/Pd and Pd/Ag samples were annealed in vacuum (*p* < 5 × 10^−3^ Pa) in the temperature range of 150–280 °C for different times.

Concentration–depth profiles were determined by measuring the intensity (cps) as a function of the sputtering time (s) using secondary neutral mass spectrometry (SNMS, INA-X, SPECS GmbH, Berlin) equipment. The intensity–sputtering time profiles were converted to concentration–depth profiles by taking into account the sensitivity factors of the elements and measuring the depth of the crater with a profilometer and by using the proportionality between the intensity and the number of sputtered particles [16,22]. The measured Pd intensities were corrected in the Ag/Pd systems. Since the difference between the atomic mass of Pd (106 amu) and Ag (107 amu) is only 1 amu, a Pd atom together with one H atoms (can be found in every vacuum chamber) can form a molecule with a mass of 107 amu. This can cause false measurements of Ag (107 amu) in the as-deposited Pd layer, as well as in the annealed samples. Thus, the Ag and Pd intensities were corrected accordingly, based on the compositions obtained in freshly deposited pure films.

## Results

**Figure 1:**
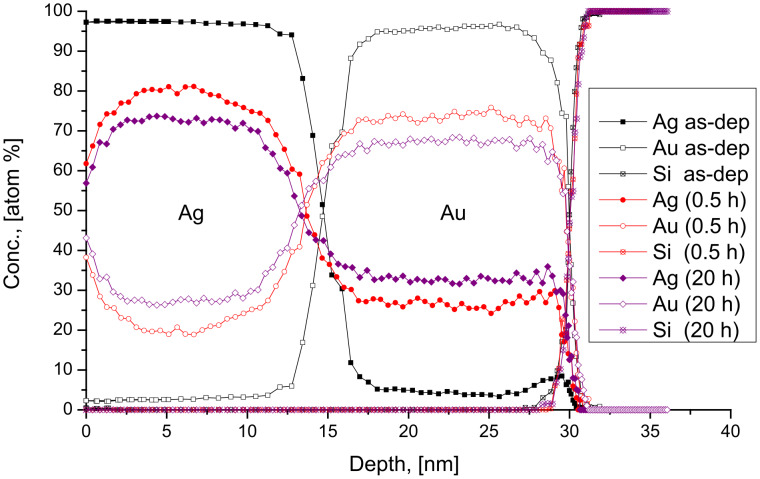
Concentration–depth profile in Ag(15 nm)/Au(15 nm) bilayer; as deposited and annealed at 170 °C.

**Figure 2:**
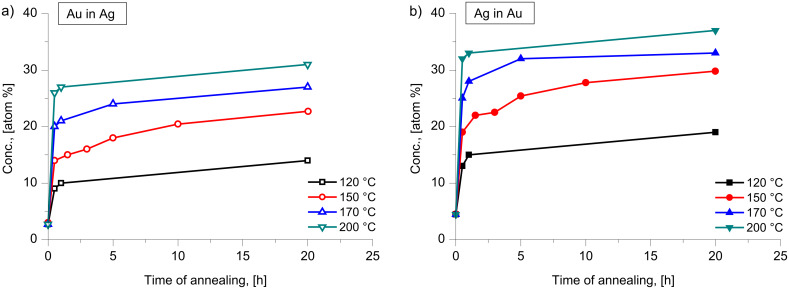
Average concentration of Au and Ag inside the Ag (a) and Au (b) layers, respectively, versus the annealing time at different temperatures.

Figure 1 shows the concentration depth profiles of the as deposited and annealed samples of Ag(15 nm)/Au(15 nm) bilayers at 170 °C, as representative example. These are very similar to curves in Figure 1a of [16], obtained at 150 °C. Figure 2a,b show the average compositions of Au in Ag and Ag in Au versus the annealing time at different temperatures, respectively. It is clear from Figure 1 and Figure 2 that there is an intensive intermixing, due to diffusion along the grain boundaries, on both sides and the average concentrations at saturation are below 50%. Furthermore, the saturation values of Au in Ag are lower than that of Ag in Au and their values increase with increasing the annealing temperature. Since, as it was also discussed in [16], our film thicknesses/grain sizes, *d*, are about two times less than double of the estimated GB migration distance (e.g., in Ag 2*L* ≈ 110 nm [6]), our *c*_sat_ values should be determined by the composition left behind the moving boundaries (our values of *c*_sat_ in Au are also not too far from the value obtained in [6]).

**Figure 3:**
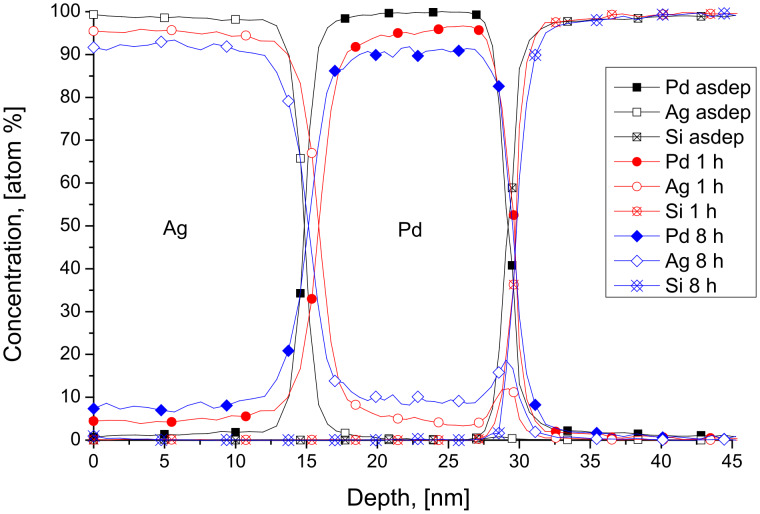
Concentration–depth profile of a Ag(15 nm)/Pd(15 nm) bilayer; as deposited and annealed at 150 °C.

**Figure 4:**
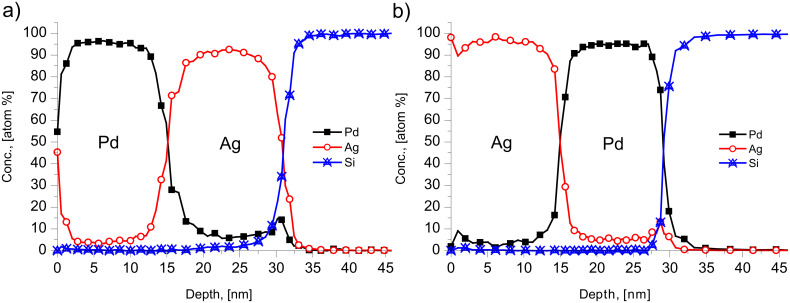
Comparison of depth profiles obtained after 8 h of heat treatment 150 °C. (a) Ag(15 nm)/Pd(15 nm)/substrate, (b) Pd(15 nm)/Ag(15 nm)/substrate. It can be seen that the reversal of the film sequence leads to the reversal of the inequality of the compositions in the centre of Pd and Ag layers.

**Figure 5:**
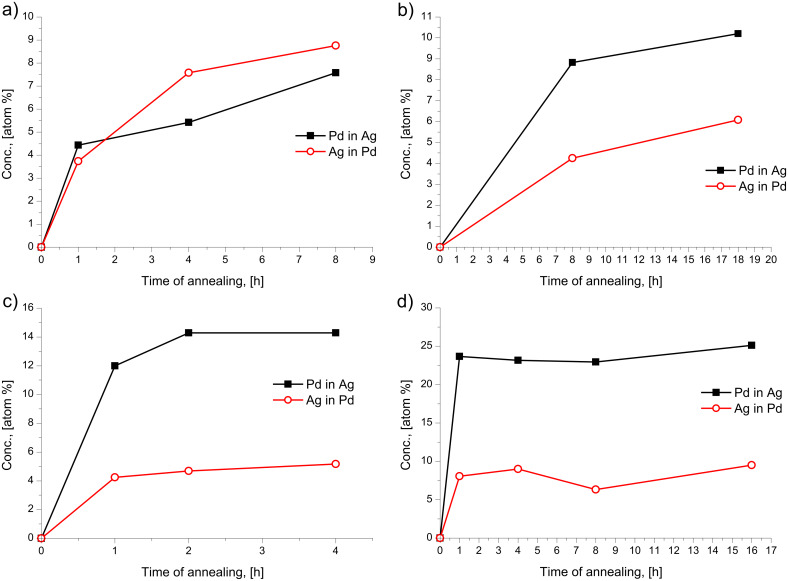
Saturation concentration of Pd and Ag inside the Ag and Pd layers, respectively, versus the annealing time in the Ag(15 nm)/Pd(15 nm) film at 150 °C (a), and in the Pd(30 nm)/Ag(30 nm) film as representative examples at 165 °C (b), 200 °C (c) and 280 °C (d).

Figure 3 shows the concentration depth profiles of the as deposited and annealed samples of Ag(15 nm)/Pd(15 nm)/substrate bilayers at 150 °C. It can be seen that the saturation value of Pd in Ag is less than that of Ag in Pd. On the other hand in the Pd(15 nm)/Ag(15 nm)/substrate system this is in reverse. Figure 4 illustrates this by comparison of the profiles of the Pd(15 nm)/Ag(15 nm) and the Ag(15 nm)/Pd(15 nm) sample obtained after 8 h of annealing at 150 °C. In addition, Figure 5 demonstrates that this reversal also remains valid in the Pd(30 nm)/Ag(30 nm)/substrate film system at different temperatures. Figure 5 shows the average compositions inside the Ag and Pd layers as a function of the annealing time at different temperatures for the Ag(15 nm)/Pd(15 nm)/substrate bilayer (Figure 5a) at 150 °C and for the Pd(30 nm/Ag(30 nm)/substrate bilayers at 165, 200 and 280 °C (Figure 5b, Figure 5c and Figure 5d, respectively). The saturation values increase with increasing annealing temperature.

**Figure 6:**
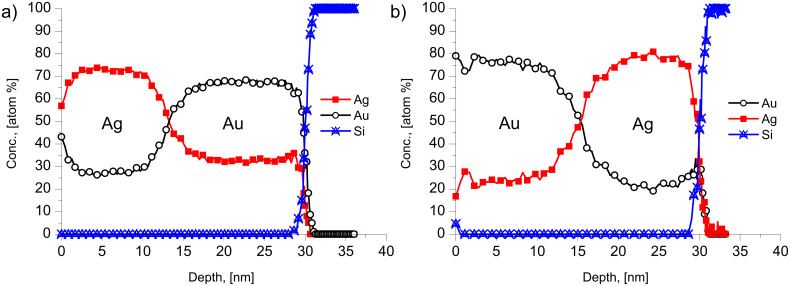
Depth profiles of Ag(15 nm)/Au(15 nm) (a) and Au(15 nm)/Ag(15 nm) (b) film systems after 20 h at 175 °C.

In the light of the above inversion effect we also compared the depth profiles obtained from Ag/Au/substrate and Au/Ag/substrate films. Figure 6 shows the results obtained after 20 h of annealing at 175 °C. It can be seen that in the case of Au/Ag sequence the compositions on both sides are practically equal to each other, reflecting that the reversal tends to decrease the expected difference in the saturation values. In addition the saturation levels are lower in the film with the Au layer outside, indicating also some stifling of the GB intermixing process.

## Discussion

First of all our results demonstrate that if the grain size is small enough (less than two times the migration distance before the DIGM slows down considerably [16]) a homogenization in bilayer films is possible on both sides, and the compositions in the DIGM zones can be followed by SNMS depth profiling.

Let us check whether the relations in Equation 1 and Equation 2 give a quantitative explanation for our experimental results or not. It can be seen that the above relations predict rather low atomic fractions if the *D*_A_/*D*_B_ ratio is large and *c*_A0_ = *c*_B0_ = 0: *c*_A_ = 0.18 and *c*_B_ = 0.05 for *D*_A_/*D*_B_ *=* 2 (using *q* = 0.1) as well as *c*_A_ = 0.10 and *c*_B_ < 0.01 for *D*_A_/*D*_B_ ≥ 20, respectively. First of all these numbers indicate that the saturation values should be larger in the slower component: Indeed they are visibly different on both sides of the Ag/Au film couples, i.e., the *c*_AginAu_ *> c*_AuinAg_ inequality was observed, in accordance with the predictions given by Equation 1 and Equation 2.

The above differences in the compositions on the two sides of the above couples also means that, using techniques having resolution of about 1 atom %, there is a hope to observe DIGM on both sides only if the *D*_A_/*D*_B_ ratio is not larger than 20. In two of our previous papers, investigating the formation and shrinkage of hollow shells in hemispherical Ag/Au and Ag/Pd systems between 430 and 470 °C [23–24], it was concluded that the *D*_Ag_/*D*_Au_ ratio should be about five times larger than unity, while *D*_Ag_/*D*_Pd_ should be larger by at least one order of magnitude than this value.

**Figure 7:**
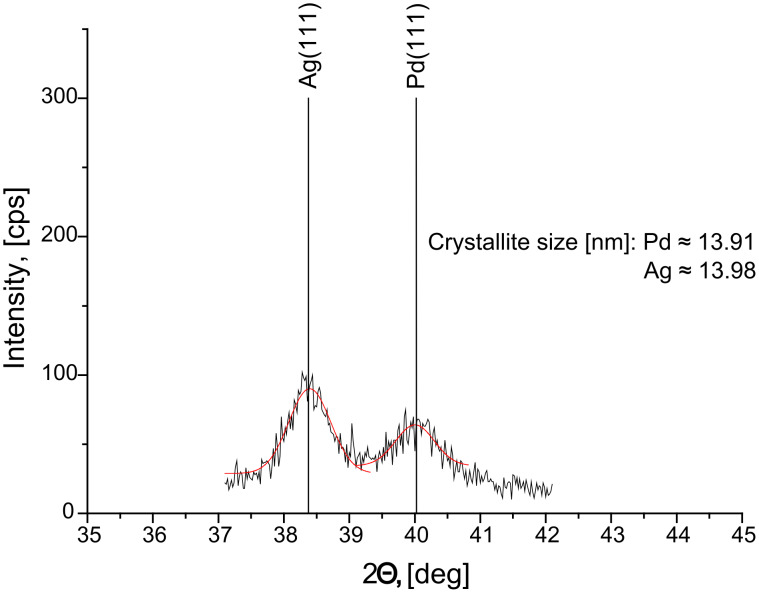
XRD results (a) obtained by using Cu Kα radiation in θ–2θ mode on Ag(15 nm)/Pd(15 nm) after heat treatment at 150 °C for 4 h. The grain sizes were determined from the half line width broadening using the Scherrer formula.

**Figure 8:**
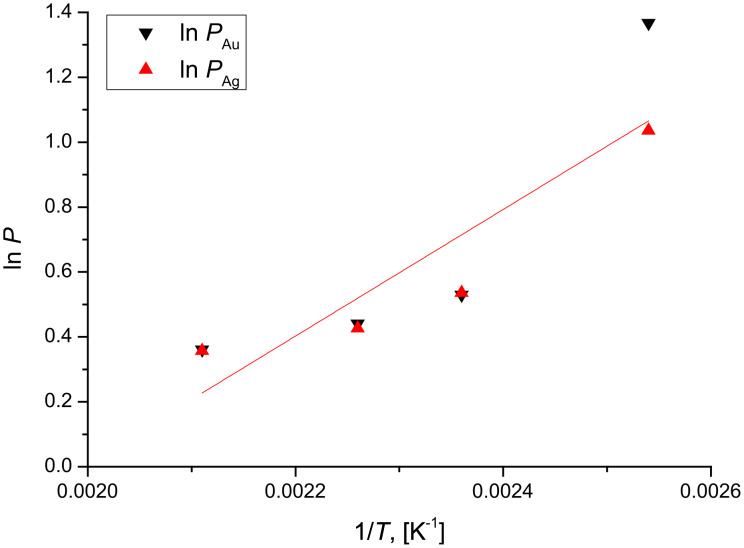
ln *P* (*P = D*_Ag_/*D*_Au_) versus 1/*T* in the Ag/Au system.

In order to get a more quantitative comparison we have to take into account that the saturation values read out from our depth profiles, *c_i,_*_sat_, are always larger than the compositions in the alloyed zone produced by DIGM, *c*′*_i,_*_sat_;

[3]
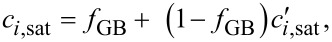


where *f*_GB_ ≈ 3δ/*d* is the grain boundary fraction and δ ≈ 0.5 nm is the grain boundary thickness. (In Equation 3 we implicitly assumed that the concentration inside the GBs is unity.) Thus the real values of *c’_i,_*_sat_ should be calculated from Equation 3. Since the grain sizes in thin films are of the same order of magnitude than the film thickness, as a first approximation, we can assume that they are equal to each other in both films in contact. In fact the TEM picture in [16] (see Figure 2 there) confirms this, and the same conclusion can be drawn from the grain size determined from the XRD line broadening in our Ag/Pd films (see Figure 7), which is *d*_Ag_ ≈ *d*_Pd_ ≈ 14 nm. Thus, in Equation 3 we can take the value of *f*_GB_ equal to 0.1 and 0.05 for film thicknesses of 15 nm and 30, respectively. Table 1 shows the real saturation compositions (as calculated by the use of Equation 3) too and the values of the *D*_Ag_/*D*_Au_ ratios, estimated using the initial conditions of *c*_A0_ *= c*_B0_ = 0. It can be seen that in the Ag/Au/substrate system these ratios, as calculated from Equation 1 and Equation 2, are between 4 and 1.5, in good accordance with the conclusions obtained in [24], and are the same in Au and Ag, i.e., they do not depend on the composition. Furthermore, although the experimental errors of the estimated ratios can be high (the uncertainty can be about 50% and 20% at the lowest and highest temperatures, respectively), the temperature dependence of this ratio has also the correct sign. Figure 8 illustrates that it increases with decreasing temperature, according to an Arrhenius function. The common slope of the two lines, gives the following value for the difference of the activation energies; *∆Q* = *Q*_Au_ − *Q*_Ag_ = 0.17 eV. This value is in very good agreement with the value obtained from the tracer diffusion data: *Q*_AuinAu_ = 0.88 eV [25] and *Q*_AginAu_ = 0.71 eV [26], i.e., ∆*Q* = 0.17 eV*.* Note that although the fitting error of Δ*Q* is ±0.04 eV, a more realistic estimate gives: *Q* = (0.2 ± 0.1) eV.

**Table 1:** Saturation values (*c_i_*_,sat_) as read out from the SNMS profiles and corrected (*c′_i_*_,sat_) according to Equation 3 in the Ag(15 nm)/Au(15 nm)/substrate system and in the Ag(15 nm)/Pd(15 nm)/substrate system at different temperatures. The estimated *D*_Au_/*D*_Ag_ ratios of the GB diffusion coefficients are also shown in (a).

Ag(15 nm)/Au(15 nm)/substrate					

*T* (°C)	*T* (K)	*c*′*_i,_*_sat_ Au in Ag	*c*′*_i_*_,sat_ Ag in Au	*D*_Ag_/*D*_Au_ in Au	*D*_Ag_/*D*_Au_ in Ag

120	393	0.055	0.122	3.9	2.8
150	423	0.141	0.221	1.7	1.7
170	443	0.188	0.255	1.6	1.5
200	473	0.233	0.300	1.4	1.4

Ag(15 nm)/Pd(15 nm)/substrate					

*T* (°C)	*T* (K)	*c_i,_*_sat_ Pd in Ag	*c_i,_*_sat_ Ag in Pd	*c*′*_i,_*_sat_ Pd in Ag	*c*′*_i,_*_sat_ Ag in Pd

150	423	≈0.10	≈0.12	≈0	≈0
200	473	≈0.10	≈0.12	≈0	≈0
250	523	0.18	0.16	0.08	0.06

The assumption that the composition inside the GB is unity can be questioned, even if our experimental observations during GB diffusion in thin film couples shows [27] that at the beginning there is a filling up of the GBs with the diffusant. In this way the diffusion along the fastest GBs results in a new (backward) source at the outer interface leading to a back diffusion along the slower GBs and the formation of a weak minimum in the centre of the films (see also the profiles in Figure 1 and Figure 4). If, as a lower limit, we assume that this composition is 0.5, then the *c*′*_i_*_,sat_ data shown in Table 1 should be increased by 0.045, which is close to the experimental uncertainty of these values. Although the value of Δ*Q* will then be smaller, it remains within the error bars given above.

**Figure 9:**
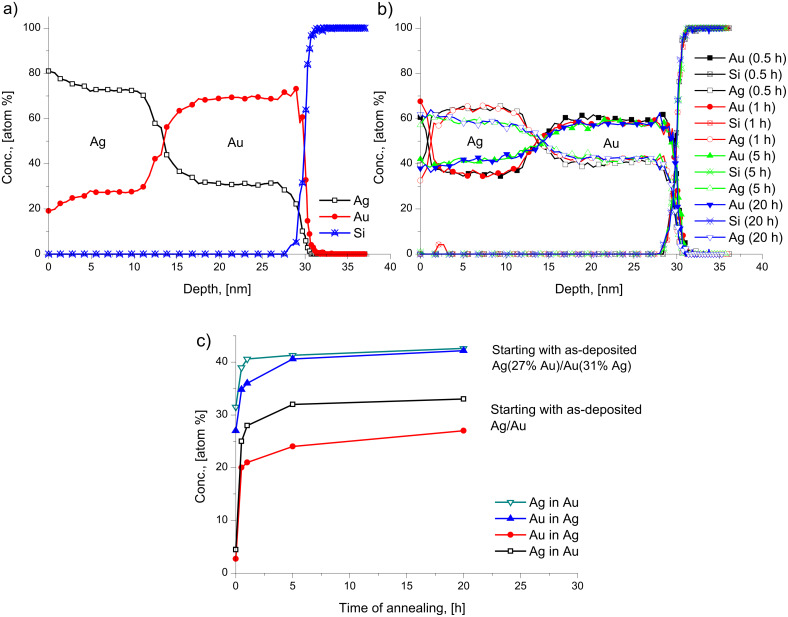
Concentration–depth profile of Ag(27% Au)/Au(31% Ag) bilayer, (a) as-deposited, (b) annealed at 170 °C for different annealing times and the average concentration of Au and Ag inside the Ag and Au layers versus annealing time at 170 °C in initially pure Ag/Au as well as Ag(27% Au)/Au(31% Ag) bilayers (c).

In principle one would expect at fixed temperatures, in systems having complete solid solubility (such as the systems investigated), that after reaching saturation, a new DIGM should begin, with the initial conditions corresponding to the first saturation values since some composition gradient is still present. Thus further increase of the average compositions in the centres of the thin films would be expected after prolonged annealing times until full homogenization is reached. That is, the formation of a solid solution corresponding to the original element ratio in the bilayer. However, we did not observe such a two- or multi-step homogenization process (see Figure 2 and Figure 5). Even prolonged heat treatments did not result in further increase of the compositions. In order to understand the reason of this, we have prepared samples with approximately the same initial compositions as the saturated sample at 170 °C. The concentration–depth profile of this Ag(27% Au)/Au(31% Ag) as-deposited sample is shown in Figure 9a. The sample was annealed at 170 °C for different times and the concentration–depth profiles are shown in Figure 9b. It is clear that there is still intermixing on both sides reaching higher saturation levels (see Figure 9c). Thus, the saturation levels obtained in initially pure samples do not correspond to the ones expected for complete intermixing and further mixing takes place only if fresh samples are produced. This should be the results of kinetic constrains, which can be attributed to two possible reasons:

1) Constrains due to stress fields caused by the initial inequality of the GB fluxes: After reaching a specific saturation, these fields do not allow for further DIGM, since they compensate the difference of the two fluxes. Indeed in the expressions for the atomic fluxes, if the stress gradients are not neglected in contrast to [4], an additional term, proportional to this gradient, appears [3–4,10].

2) Constrains due to a finite-size effect: For thin films, the second derivative of the composition along the GBs should gradually decrease (because of the reflections from the film boundaries) and thus the interface velocity can also gradually decay, since it is proportional to the second derivative of the composition (see Equation 3 in [4]).

Nevertheless we can check the validity of Equation 1 and Equation 2 in predicting the saturation values in the second step. Using again Equation 3 for the initial conditions: *c*′_Ag0_ = 0.23 (*c*_Ag0_ = 0.31) and *c*′_Au0_ = 0.19 (*c*_Au0_ = 0.27) as well as taking as an average value *D*_Ag_/*D*_Au_ = 2.0 (Table 1a) we obtain, from the profiles shown in Figure 9c: *c*′_Ag_ = 0.39 as well as c′_Au_ = 0.31, which should be compared with values *c*′_Ag_ = 0.37 as well as c′_Au_ = 0.36 (calculated from the values *c*_Ag_ = 0.43 and *c*_Au_ = 0.42 read out from Figure 9c). It can be seen that the agreement is very good.

In contrast to the Ag/Au/substrate systems, where the agreement with the step model of GB motion during DIGM is very good, there is a qualitative agreement only for the results obtained in Ag(15 nm)/Pd(15 nm)/substrate films. Qualitative agreement means that the larger saturation value of Ag in Pd (as compared to that of the Pd in Ag) is in accordance with the fact that *D*_Ag_/*D*_Pd_ > 1 [24]. On the other hand reversing the film sequence the inequality of the saturation values reversed too: this is in contrast to the expectations based on Equation 1 and Equation 2. Furthermore, estimations such as those for the Ag/Au films (Table 1), cannot be made for the Ag(15 nm)/Pd(15 nm)/substrate films since the differences between the *c_i_*_,sat_ and *c*′*_i,_*_sat_ values lie within the error bars. The only conclusion we can draw is that all the real saturation compositions are less than 0.1 indicating that the *D*_Ag_/*D*_Pd_ ratio should be larger than 20, which is in line with the conclusions of [24].

The failure of the predictions in films with inverted layer sequence (Au/Ag/substrate and Pd/Ag/substrate bilayers) can be the effect of stress: for *D*_Ag_ > *D*_Au_ and *D*_Ag_ > *D*_Pd_ it is expected that the inequality of the GB fluxes will lead to tensile stress in Ag and compressive stress in Au and Pd (for instance, during DIGM Au expands and Ag shrinks [5–6,10]). This can develop a stress gradient that diminishes the difference of the fluxes. However, this effect alone would not be enough to reverse the resultant atomic flow. The effect of the substrate can also have an influence on the process: The sign of the stress gradient due to this effect can be the same in both films and in this case it can have an influence on the DIGM (this correction term would have the same sign for both, opposite, GB fluxes). The substrate effect alone, in line with the results of [6] where the GB velocity was only slightly different for DIGM in the Au layers (in contact with Ag) on solid substrates or in free-standing films, also would not be enough to reverse the composition. On the other hand, the combined effect of the presence of the substrate and the change of the film sequence, can lead to significant changes in the atomic GB fluxes. The concrete situation (and stress distributions) can be complicated enough to get a quantitative prediction here. Thus, further theoretical and experimental investigations are desired to understand the observations described above.

Finally it is worth to mention the recent work on Ag/Pd system [28], in which a similar cold homogenization of free standing nanostructured Ag/Pd films (with grains of about 5–10 nm and individual film thicknesses between 20 and 60 nm) at similar and slightly higher temperatures (between 180 and 350 °C) was investigated by in situ TEM heating technique and electron diffraction methods. It was concluded, from the detection of the positions of the diffraction lines and their intensity, that mixing of components occurred already during the room temperature deposition of Ag on Pd. Furthermore, the change of the half width of the (220) reflexion peaks of Pd and Ag was used as a measure of intermixing. From these, and from direct TEM investigations, it was concluded that the formation of a solid solution, with a composition corresponding to the initial thickness ratio in the bilayer, took place inside the nanograins without significant changes in the film morphology. Significant grain growth was observed only above 500 °C. From the estimation of the time necessary for homogenization, τ, using the relation *x*^2^ = *D*τ and taking *x* = 5–10 nm, *D* ≈ 10^−17^–10^−18^ m^2^/s was obtained for the effective diffusion coefficient at 280 °C. Since this value is about eight orders of magnitude (!) larger than the bulk diffusion coefficient of Pd in Ag at 280 °C [26] and about two to three orders of magnitude smaller than the Ag GB self-diffusion [29] at the same temperature, they concluded that the observed phenomena can be explained by activated bulk diffusion due to a structure of nano-grains with an increased number of defects. We suggest a different explanation, because it is difficult to offer any mechanism for an increase of the bulk diffusion coefficient of many orders of magnitude and also because such an increase does not offer an explanation for the effect of the reversal of the film thickness. In addition, the good agreement with the model predictions in the Ag/Au system also favours the explanation offered by us. Furthermore, it is worth underlining one important difference between our observations and those reported in [28]: In our films rather moderate levels of saturation are reached (see Figure 3, Figure 4 and Figure 5), while in [28] it was clearly declared that they reached the full homogenization (with about 47% of Ag in Pd) corresponding to the ratio of film thicknesses. This point still preserves attention for further investigations and can also be related (at least at higher temperatures) to the contribution of the bulk diffusion to DIGM. Indeed in the model explanation of Hillert [2] and Penrose [8] some bulk diffusion should take place ahead the moving boundary and thus at elevated temperatures Equation 1 and Equation 2 can fail, and the composition can be determined be the relation derived in [2]. Furthermore, at higher temperatures the diffusion-induced recrystallization (DIR) can also play a role in cold alloying. It was illustrated in [30], that at 450 °C in a 100 nm Ag/200 nm Pd/200 nm Ag/100 nm Pd multilayered film, using EDX maps of Ag and Pd concentrations that the Pd layer has considerably shrunk in thickness, but has preserved a composition close to pure Pd. By contrast, the Ag layer appeared to be intermixed to a Pd content of about 35 atom % on average. At the same time the mixing was accompanied by severe microstructural transformations and the generation of new grains within the diffusion zone with a preferred initial composition of 24.5 atom % Pd. Note that a similar process at low temperature would lead to a similar profile like to the one shown in Figure 4b.

## Conclusion

It is illustrated that the compositions in the DIGM zone can be determined by depth profiling in nanostructured thin films, using SNMS as depth-profiling technique. The results can be described with the help of relations provided by the microscopic model for GB diffusion-induced GB motions via a step mechanism in the Ag/Au/substrate system:

1. The saturation compositions in the slower component, in agreement with Equation 1 and Equation 2, are always larger than in the faster one, in the Ag/Au/substrate system.
2. The temperature dependence of the saturation values are also in line with the predictions of the above model and reflect the temperature dependence of the ratios of the GB diffusion coefficients of the two components,
3. DIGM stops after the first step of homogenization (i.e., after reaching the composition predicted by the formation of the first DIGM zones), although some composition gradient is still present in the thin film.
4. It was demonstrated that the effect in point iii) can be explained by some kind of kinetic constrains developed by the GB diffusion itself (stress accumulation/decrease of the composition gradient due to finite size effects).

The results in the Ag/Pd/substrate system showed a qualitative agreement with the above model, indicating that here the *D*_Ag_/*D*_Pd_ ratio is larger than 20 and thus the compositions in the DIGM zone are of the same order of magnitude as the average compositions due to filling up of the GB area. On the other hand, the reversal of the film sequence in both systems led to a conflict with the saturation values suggested by Equation 1 and Equation 2: Even the predicted inequality of the saturation values cannot be explained by them. Possible different roles of the stress gradients, created by the GB intermixing is offered as possible explanation.

## References

[1] Hillert M, Purdy G R (1978). Acta Metall.

[2] Hillert M (1983). Scr Metall.

[3] Shewmon P G (1981). Acta Metall.

[4] Balluffi R W, Cahn J W (1981). Acta Metall.

[5] Hwang J C M, Pan J D, Balluffi R W (1979). J Appl Phys.

[6] Pan J D, Balluffi R W (1982). Acta Metall.

[7] Handwerker C A, Cahn J W (1987). Mater Res Soc Symp Proc.

[8] Penrose O (2004). Acta Mater.

[9] Schmitz G, Baither D, Kasprzak M, Kim T H, Kruse B (2010). Scr Mater.

[10] Klinger L, Rabkin E (2011). Acta Mater.

[11] Kasprzak M, Baither D, Schmitz G (2011). Acta Mater.

[12] Tu K N (1977). J Appl Phys.

[13] den Broeder F J A (1985). Thin Solid Films.

[14] Shenouda S S, Langer G A, Katona G L, Daróczi L, Csik A, Beke D L (2014). Appl Surf Sci.

[15] Molnár G, Erdélyi G, Langer G, Beke D L, Csik A, Katona G L, Daróczi L, Kis-Varga M, Dudás A (2013). Vacuum.

[16] Shenouda S S, Katona G L, Langer G A, Daróczi L, Beke D L (2015). Mater Lett.

[17] Tynkova A, Katona G L, Langer G A, Sidorenko S I, Voloshko S M, Beke D L (2014). Beilstein J Nanotechnol.

[18] Katona G L, Vladymyrskyi I A, Makogon I M, Sidorenko S I, Kristály F, Daróczi L, Csik A, Liebig A, Beddies G, Albrecht M (2014). Appl Phys A.

[19] Beke D L, Langer G A, Molnár G, Erdélyi G, Katona G L, Lakatos A, Vad K (2013). Philos Mag.

[20] Molnár G, Katona G L, Langer G A, Csík A, Chen Y C, Beke D L (2015). Mater Res Express.

[21] den Broeder F J A, Nakahara S (1983). Scr Metall.

[22] Wucher A, Oeschner H, Fresenius Z (1989). Anal Chem.

[23] Glodán G, Cserháti C, Beszeda I, Beke D L (2010). Appl Phys Lett.

[24] Glodán G, Cserháti C, Beke D L (2012). Philos Mag.

[25] Gupta D (1973). J Appl Phys.

[26] Klotsman S M, Arkhipova N K, Timofejev A N, Traktenberg I S (1968). Phys Met Metallogr.

[27] Makovec A, Erdélyi G, Beke D L (2012). Thin Solid Films.

[28] Krysthal A P, Bogatyenko S I, Sukhov R V, Minenkov A A (2014). Appl Phys A.

[29] Adams J B, Foiles S M, Wolfer W G (1989). J Mater Res.

[30] Baither D, Kim T H, Schmitz G (2008). Scr Mater.

